# Moderation of the real-world effectiveness of smoking cessation aids by mental health conditions: A population study

**DOI:** 10.1371/journal.pmen.0000007

**Published:** 2024-06-04

**Authors:** Sarah E. Jackson, Leonie Brose, Vera Buss, Lion Shahab, Deborah Robson, Jamie Brown

**Affiliations:** 1 Department of Behavioural Science and Health, University College London, London, United Kingdom; 2 SPECTRUM Consortium, London, United Kingdom; 3 Addictions Department, Institute of Psychiatry, Psychology and Neuroscience, King’s College London, London, United Kingdom; BronxCare Health System, UNITED STATES

## Abstract

**Objective:**

To examine whether the real-world effectiveness of popular smoking cessation aids differs between users with and without a history of mental health conditions.

**Design:**

Nationally-representative cross-sectional survey conducted monthly between 2016–17 and 2020–23.

**Setting:**

England.

**Participants:**

5,593 adults (2,524 with a history of ≥1 mental health conditions and 3,069 without) who had smoked regularly within the past year and had attempted to quit at least once in the past year.

**Main outcome measures:**

The outcome was self-reported abstinence from quit date up to the survey. Independent variables were use of the following cessation aids during the most recent quit attempt: prescription nicotine replacement therapy (NRT), NRT over-the-counter, varenicline, bupropion, vaping products, face-to-face behavioural support, telephone support, written self-help materials, websites, hypnotherapy, Allen Carr’s Easyway, heated tobacco products, and nicotine pouches. The moderator was history of diagnosed mental health conditions (yes/no). Covariates included sociodemographic characteristics, level of cigarette addiction, and characteristics of the quit attempt.

**Results:**

Relative to those without, participants with a history of mental health conditions were significantly more likely to report using vaping products (38.8% [95%CI 36.7–40.8] vs. 30.7% [28.9–32.5]), prescription NRT (4.8% [3.9–5.7] vs. 2.7% [2.1–3.3]), and websites (4.0% [3.2–4.8] vs. 2.2% [1.6–2.7]). Groups did not differ significantly in their use of other aids. After adjusting for covariates and use of other cessation aids, those who used vaping products (OR = 1.92, 95%CI 1.61–2.30), varenicline (OR = 1.88, 95%CI 1.19–2.98), or heated tobacco products (OR = 2.33, 95%CI 1.01–5.35) had significantly higher odds of quitting successfully than those who did not report using these aids. There was little evidence that using other cessation aids increased the odds of successful cessation, or that the user’s history of mental health conditions moderated the effectiveness of any aid.

**Conclusions:**

Use of vaping products, varenicline, or heated tobacco products in a quit attempt was associated with significantly greater odds of successful cessation, after adjustment for use of other cessation aids and potential confounders. There was no evidence to suggest the effectiveness of any popular cessation aid differed according to the user’s history of mental health conditions.

## Introduction

Tobacco smoking remains a leading cause of preventable illness and premature mortality in England [1]. Relative to the general population, people with mental health conditions are more likely to smoke, smoke more heavily, and show greater signs of dependence [2–5]. They are also at increased risk of tobacco-related morbidities, including cardiovascular disease [6, 7], which causes them to have substantially lower life expectancy [8, 9]. Quitting smoking can reduce these risks [10]. A range of smoking cessation aids have been found to increase successful smoking cessation in randomised controlled trials (RCTs) [11–14] and in real-world settings [15–24]. Understanding whether and, if so, how far their effectiveness differs between people with and without mental health conditions can help health professionals and patients to make informed choices around the use of aids for smoking cessation.

In England, a comprehensive range of smoking cessation medications and behavioural support are available [25]. Pharmacological aids include nicotine replacement therapy (NRT), which is available free of charge on prescription or can be bought over-the-counter (OTC), and varenicline (Champix) and bupropion (Zyban) which are only available on prescription. The supply of Champix, the most effective of these [14, 23, 24], was disrupted in 2021 due to nitrosamine impurities found by its supplier, Pfizer [26]. The supply of Zyban was disrupted in late 2022 due to similar concerns about nitrosamine impurities found by its supplier, GSK [27]. Both medications remain unavailable as of March 2024. Generic versions of varenicline are available in other countries and cytisine, a drug with similar properties to varenicline, is licensed [28] and has begun to be supplied in England since January 2024.

Vaping products (often referred to as e-cigarettes) and nicotine pouches are available from specialist ‘vape shops’, supermarkets, smaller convenience stores, and online [29]. Heated tobacco products have been available in the UK since 2016 but their efficacy for smoking cessation is uncertain [30]. Smokers also have access to free dedicated stop smoking services, which offer behavioural support, pharmacotherapy, and in some cases, vaping products [31]. Telephone support is available via a free Smokefree National Helpline, and websites offer information on quitting, other forms of support available, and how to access them. Other behavioural treatments, including hypnotherapy and Allen Carr’s Easyway method (a single-session pharmacotherapy-free behavioural programme) [32, 33], are provided by private companies.

Around one in two attempts to stop smoking in England involves the use of at least one of these cessation aids [34]. Vaping products are most commonly used (~30% of quit attempts), followed by NRT available OTC (~10%) and medications obtained on prescription (NRT, varenicline, or bupropion; ~5%) [34].

It is possible that the effectiveness of these smoking cessation aids may differ between people with and without mental health conditions [35]. People with mental health conditions may experience stronger reinforcing effects of nicotine, more severe withdrawal symptoms when they try to quit, and greater cessation fatigue (being tired of trying to stop smoking) [36]. As such, it is possible that they may benefit more from cessation aids that mimic the effect of nicotine (e.g., varenicline) or provide an alternative source of nicotine (e.g., NRT, vaping products, nicotine pouches) and less from other cessation aids (e.g., forms of behavioural support). On the other hand, people with mental health conditions may be less likely to adhere to treatments [37], causing effectiveness to be lower.

To our knowledge, just two large experimental studies have investigated whether the effectiveness of smoking cessation treatments is moderated by a person’s mental health status. These have focused on varenicline, bupropion, and NRT. One large RCT (‘EAGLES’) compared varenicline and bupropion with nicotine patch and placebo and showed similar efficacy of these medications for smokers with and without psychiatric disorders [38]. However, a recent secondary analysis of another RCT that compared the effectiveness of bupropion and varenicline reported a slightly different pattern of results [39]. While varenicline was associated with similar quitting outcomes for smokers with depressive symptoms than those without, bupropion appeared to be *less* effective as a smoking cessation aid for those with depressive symptoms [39]. Further research is required on these and other cessation aids. Observational data can shed light on any differences in treatment effectiveness in real-world settings [40].

Using data from the Smoking Toolkit Study, a large, nationally-representative survey of adults in England, this study aimed to comprehensively examine whether the real-world effectiveness of popular smoking cessation aids differs between users with and without mental health conditions. Data on smoking status in relation to history of mental health conditions have been published elsewhere [2], so this paper focused specifically on the use and effectiveness of different cessation aids among those attempting to quit smoking. Specifically, we aimed to address the following research questions:

1. To what extent does a history of one or more diagnosed mental health conditions moderate associations between use (vs. non-use) of various cessation aids in a quit attempt and chances of success?
2. Are any moderating effects similar for those with a single mental health condition and those with multiple mental health conditions?

## Materials and methods

### Pre-registration

The study protocol and analysis plan were pre-registered on Open Science Framework (osf.io/5xubc). We made one amendment. We had planned to calculate Bayes factors for non-significant interactions based on an expected effect size of OR = 1.5 in the observed direction (i.e., OR = 1.5 for observed ORs >1 and OR = 0.67 for observed ORs <1). Instead, we calculated Bayes factors in both directions, to offer more insight into whether the data suggested a given aid was more or less effective for people with a history of mental health conditions than those without.

### Design

This was an observational study using data from the Smoking Toolkit Study; a nationally-representative monthly cross-sectional survey of adults (≥16 years) in England [41]. The study uses a hybrid of random probability and simple quota sampling to select a new sample of approximately 1,700 adults aged ≥16 years each month. Comparisons with other national surveys and sales data indicate that the survey obtains nationally-representative estimates for key variables including sociodemographic characteristics, smoking prevalence, and cigarette consumption [41, 42]. The Smoking Toolkit Study is coordinated by this study’s authors at University College London (PI Jamie Brown).

Data collection for the Smoking Toolkit Study began in November 2006 and the study continues to collect data from a new sample each month. Up to February 2020, the survey was conducted via face-to-face computer-assisted interviews. However, social distancing restrictions introduced during the Covid-19 pandemic meant that no data were collected in March 2020, and data from April 2020 onwards have been collected via telephone interviews. The telephone interviews use a similar sampling and weighting approach as the face-to-face interviews and data collected via the two modalities show good comparability [43–45]. Data were not collected from 16 and 17 year olds between April 2020 and December 2021.

While a core set of questions is included in each monthly survey, other variables are only assessed in certain waves, depending on availability of competitive research funding. Questions on mental health have been collected in two periods: January 2016-December 2017 and October 2020-June 2023. We used data from participants surveyed in these periods. We selected participants aged ≥18 years who:

- smoked cigarettes (including hand-rolled) or any other tobacco product (e.g., pipe or cigar) daily or occasionally at the time of the survey or during the past year; and
- reported having made at least one serious quit attempt in the past year.

### Ethics statement

Ethical approval was provided by the UCL Research Ethics Committee (0498/001). Participants provided informed verbal consent to take part in the study, and all methods are carried out in accordance with relevant regulations. The data are not collected by UCL and are anonymised when received by UCL.

### Measures

#### Outcome: Successful smoking cessation

The outcome variable was self-reported continuous abstinence from the start of the most recent quit attempt up to the time of survey. Respondents were asked ‘How long did your most recent quit attempt last before you went back to smoking?’ Responses were coded 1 for those who responded that they were still not smoking and 0 otherwise.

#### Exposures: Use of cessation aids

Independent variables were self-reported use or not (dummy coded) of the following smoking cessation aids in the most recent quit attempt: prescription NRT (available in England from prescribing health professionals, including advisors at specialist stop smoking services); NRT available OTC; varenicline; bupropion; vaping products; face-to-face behavioural support; telephone support; written self-help materials; websites; hypnotherapy; Allen Carr’s Easyway; heated tobacco products; and nicotine pouches.

Respondents were asked to indicate all that apply, and data for each were coded 1 if chosen and 0 if not. Heated tobacco products were included in the list of response options from April 2016 and nicotine pouches from June 2021; given the low prevalence of use of these products [46, 47], we imputed missing values as 0 for participants surveyed before the response options were introduced.

#### Moderator: History of mental health conditions

Diagnosed mental health conditions were assessed with the question: ‘Since the age of 16, which of the following, if any, has a doctor or health professional ever told you that you had?’ followed by a list of ICD-10 recognised conditions: depression; anxiety; obsessive compulsive disorder; panic disorder or a phobia; post-traumatic stress disorder; psychosis; personality disorder; attention deficit hyperactivity disorder; an eating disorder; alcohol misuse or dependence; drug use or dependence; and problem gambling. Between 2020 and 2023, this list also included: autism or autism spectrum disorder; and bipolar disorder.

For our primary analysis (RQ1), those who reported any of these diagnoses were coded 1, else they were coded 0 (including those who did not respond, responded ‘don’t know’, or ‘prefer not to say’).

For our secondary analysis (RQ2), we subdivided the group reporting mental health diagnoses to create a three-level variable: no history of mental health conditions (coded 0), single mental health condition (1 diagnosis; coded 1), and multiple mental health conditions (≥2 diagnoses; coded 2), given previous evidence showing stronger associations with smoking outcomes among those with multiple conditions [2].

#### Covariates

Covariates included a range of sociodemographic characteristics, level of cigarette addiction, and variables relating to the most recent quit attempt.

Sociodemographic covariates included age, gender, and occupational social grade (ABC1, which includes managerial, professional and intermediate occupations, vs. C2DE, which includes lower supervisory and technical occupations, semi‐routine and routine occupations, never worked and long‐term unemployed).

Level of cigarette addiction was assessed by asking participants to self-report ratings of the strength of urges to smoke over the last 24 hours (not at all (coded 0), slight (1), moderate (2), strong (3), very strong (4), extremely strong (5)). This question was also coded ‘0’ for smokers who respond ‘not at all’ to the (separate) question ‘How much of the time have you spent with the urge to smoke?’ [48]. This validated measure has similar predictive value as the Fagerström Test of Cigarette Dependence and the Heaviness of Smoking Index in for cessation [49].

The characteristics of the most recent quit attempt included time since the quit attempt started (<1 month, 1–6 months, >6 months), the number of prior quit attempts in the past year (1, 2, 3 or ≥4), whether the quit attempt was planned, and whether the respondent cut down first or stopped abruptly.

The month and year of survey were also included to account for seasonal variation in quit attempts (e.g., in January or ‘Stoptober’ [50, 51]) and changes in the availability and regulation of different smoking cessation aids over the study period. We also adjusted for the mode of data collection with a variable coded 0 up to February 2020 (when data were collected face to face) and 1 from April 2020 onwards (when data were collected via telephone).

### Statistical analysis

The Smoking Toolkit Study uses survey weights to adjust data so that the sample matches the demographic profile of England on age, social grade, region, housing tenure, ethnicity and working status within sex [41]. The following analyses used weighted data. Missing values were excluded on a per-analysis basis.

We calculated the proportion and 95% confidence interval (CI) of smokers with and without mental health conditions reporting using each cessation aid in the most recent quit attempt, and the quit success rate among users of each aid. We also provided descriptive data on the proportion reporting using each cessation aid and the overall quit success rate separately for each individual mental health condition.

We used logistic regression to analyse associations between self-reported abstinence (abstinent yes vs. no) and use of different smoking cessation aids (use of a specific aid vs. no use of that specific aid), adjusting for mental health status, covariates, and use of other cessation aids (baseline model). We repeated the baseline model with the addition of the two-way interaction between mental health diagnoses (0 vs ≥1 mental health conditions) and each cessation aid in turn.

To explore any differences between those with single and multiple mental health diagnoses, we reran the interactions using a 3-level mental health variable (0, 1, ≥2 mental health conditions).

We calculated Bayes factors using an online calculator (bayesfactor.info) to aid in the interpretation of non-significant interactions with mental health diagnoses. These enabled us to examine whether these associations could best be characterised as evidence of no effect, evidence of an effect, or whether data were insensitive to detect an effect [52, 53]. Alternative hypotheses were represented by half-normal distributions and the expected effect size set to OR = 1.5 in the observed direction (OR = 1.5 where the observed OR was >1 and OR = 0.67 when the observed OR was <1) [23].

## Results

Of 95,952 participants surveyed in eligible waves, 17,394 (18.1%) reported smoking in the past year, of whom 5,741 (33.0%) attempted to stop smoking in the past year. We excluded 148 participants with missing data on mental health conditions (there were no missing data on use of cessation aids), leaving a final sample for analysis of 5,593 participants.

Just under half (45.1% weighted) of participants reported having ever been diagnosed with a mental health condition; 16.8% reported a single mental health condition and 28.3% multiple conditions. **Table 1** summarises the characteristics of participants with and without a history of mental health conditions. **S1 Table** presents corresponding data for those reporting single and multiple mental health conditions. Relative to those without, participants reporting a history of mental health conditions were more likely to be younger, identify as women or non-binary, and come from less advantaged social grades. They also reported a higher level of cigarette addiction, on average, but there were no notable differences in the characteristics of their most recent quit attempt.

**Table 1:** Weighted sample characteristics.

	All participants	No history of a MHC	One or more MHCs
*Unweighted N*	*5593*	*3069*	*2524*
**Sociodemographic characteristics**			
Age (years), %			
16–24	21.4 (20.2–22.6)	19.4 (17.9–21.0)	23.8 (22.0–25.6)
25–34	27.7 (26.4–29.0)	26.5 (24.7–28.3)	29.2 (27.3–31.2)
35–44	18.4 (17.3–19.6)	18.4 (16.9–20.0)	18.4 (16.8–20.2)
45–54	15.0 (14.0–16.0)	15.7 (14.4–17.1)	14.1 (12.8–15.6)
55–64	10.4 (9.6–11.2)	11.0 (10.0–12.2)	9.5 (8.5–10.8)
≥65	7.2 (6.5–7.9)	9.0 (8.1–10.0)	4.9 (4.1–5.8)
Gender, %			
Man	52.2 (50.8–53.6)	59.8 (57.9–61.7)	42.9 (40.8–45.0)
Woman	47.0 (45.6–48.4)	40.0 (38.1–41.8)	55.6 (53.5–57.7)
In another way^1^	0.8 (0.6–1.1)	0.2 (0.1–0.5)	1.6 (1.2–2.1)
Social grade, %			
ABC1 (more advantaged)	43.2 (41.8–44.6)	46.0 (44.1–47.8)	39.8 (37.9–41.8)
C2DE (less advantaged)	56.8 (55.4–58.2)	54.0 (52.2–55.9)	60.2 (58.2–62.1)
**Level of cigarette addiction**			
Strength of urges to smoke, mean (SD)	1.7 (1.3)	1.5 (1.2)	1.8 (1.3)
**Features of the most recent quit attempt**			
Time since quit attempt started, %			
< 1 month	15.6 (14.6–16.6)	16.0 (14.6–17.4)	15.1 (13.6–16.6)
Between 1 and 6 months	46.5 (45.1–47.9)	45.5 (43.6–47.4)	47.7 (45.6–49.9)
> 6 months	37.9 (36.6–39.3)	38.5 (36.7–40.4)	37.2 (35.2–39.3)
Number of past-year quit attempts, %			
1	64.7 (63.3–66.0)	65.0 (63.1–66.8)	64.4 (62.3–66.4)
2	20.2 (19.1–21.4)	20.3 (18.8–21.9)	20.1 (18.4–21.8)
3	7.7 (7.0–8.5)	7.2 (6.3–8.3)	8.2 (7.2–9.4)
≥4	7.4 (6.7–8.2)	7.5 (6.5–8.6)	7.3 (6.3–8.5)
Quit attempt was unplanned, %	56.6 (55.1–58.0)	56.4 (54.5–58.4)	56.7 (54.6–58.8)
Quit attempt was abrupt, %	53.4 (52.0–54.8)	53.1 (51.1–55.0)	53.8 (51.7–55.9)

MHC, mental health condition.

Data are weighted to match the adult population in England.

Note: There were some missing data for the following variables: age *n* = 3, sex *n* = 9, strength of urges to smoke *n* = 77, time since quit attempt started *n* = 69, quit attempt was unplanned *n* = 179, quit attempt was abrupt *n* = 80. Valid percentages are shown.

^1^ This group was excluded from the regression analyses (which adjust for gender) in **Table 3** due to low numbers.

Corresponding data with history of MHCs coded as 0, 1, or ≥2 MHCs are provided in **S1 Table**.

**Table 2** summarises use of cessation aids by participants’ history of mental health conditions. Participants with a history of mental health conditions were significantly more likely to report using one or more cessation aids (58.9%) than those without (52.9%) and to report using multiple aids (12.0% vs. 7.3%). Among those with and without a history of mental health conditions, vaping products were the most commonly used aid (38.8% and 30.7%, respectively), followed by NRT available over-the-counter (16.7% and 17.8%). All other aids were used by <5% of participants. Relative to those without, participants with a history of mental health conditions were more likely to report using vaping products, prescription NRT, and websites. Use of other aids did not differ significantly between groups. There was no significant difference in the prevalence of use of any cessation aid between those with single versus multiple mental health conditions (**S2 Table**).

**Table 2:** Use of cessation aids in the most recent quit attempt by history of mental health conditions.

	% (95% CI)		
	All participants	No history of a MHC	One or more MHCs
**Use in the most recent quit attempt of…** ^***1***^			
Vaping products	34.3 (33.0–35.7)	30.7 (28.9–32.5)	38.8 (36.7–40.8)
NRT available over-the-counter	17.3 (16.2–18.4)	17.8 (16.4–19.3)	16.7 (15.1–18.2)
Prescription NRT	3.7 (3.1–4.2)	2.7 (2.1–3.3)	4.8 (3.9–5.7)
Varenicline	3.5 (3.0–4.0)	3.5 (2.8–4.3)	3.4 (2.7–4.2)
Websites	3.0 (2.5–3.5)	2.2 (1.6–2.7)	4.0 (3.2–4.8)
Face-to-face behavioural support	2.2 (1.8–2.7)	1.8 (1.3–2.3)	2.8 (2.1–3.4)
Allen Carr’s Easyway	1.3 (0.9–1.6)	1.2 (0.7–1.6)	1.4 (0.9–1.9)
Written self-help materials	0.9 (0.6–1.2)	1.1 (0.6–1.5)	0.7 (0.4–1.1)
Nicotine pouches	0.9 (0.6–1.2)	0.8 (0.4–1.1)	1.0 (0.6–1.5)
Telephone support	0.8 (0.5–1.0)	0.7 (0.4–1.0)	0.9 (0.5–1.3)
Heated tobacco products	0.8 (0.5–1.0)	0.7 (0.3–1.0)	0.9 (0.5–1.2)
Hypnotherapy	0.7 (0.5–0.9)	0.6 (0.3–0.9)	0.8 (0.4–1.1)
Bupropion	0.5 (0.3–0.7)	0.4 (0.2–0.6)	0.6 (0.2–0.9)
**Number of these aids used**			
0 (unaided quitting)	44.4 (43.0–45.8)	47.1 (45.2–49.0)	41.1 (39.1–43.2)
1	45.8 (44.4–47.2)	44.9 (43.0–46.8)	46.9 (44.8–49.0)
2	7.3 (6.6–8.1)	6.2 (5.3–7.2)	8.7 (7.6–10.0)
3 or more	2.5 (2.1–3.0)	1.9 (1.4–2.5)	3.3 (2.6–4.1)
**Quit success among those who used…**			
Vaping products	26.1 (23.9–28.2)	24.8 (21.7–27.8)	27.4 (24.3–30.4)
NRT available over-the-counter	19.8 (17.1–22.6)	19.2 (15.6–22.8)	20.7 (16.5–24.9)
Prescription NRT	21.0 (14.6–27.4)	21.6 (10.5–32.8)	20.6 (12.9–28.2)
Varenicline	22.0 (15.3–28.6)	21.5 (12.0–30.9)	22.6 (13.3–31.9)
Websites	26.0 (18.5–33.6)	20.4 (9.5–31.3)	29.7 (19.4–40.0)
Face-to-face behavioural support	22.7 (14.5–31.0)	20.0 (8.0–32.0)	25.0 (13.5–36.4)
Allen Carr’s Easyway	15.2 (5.9–24.6)	13.3 (1.7–25.0)	17.2 (2.2–32.1)
Written self-help materials	14.5 (3.9–25.1)	10.9 (0–23.5)	20.8 (0.8–40.8)
Nicotine pouches	24.7 (11.4–38.0)	18.2 (1.7–34.7)	30.5 (9.9–51.0)
Telephone support	31.5 (16.7–46.3)	36.4 (12.6–60.1)	27.3 (8.0–46.6)
Heated tobacco products	27.4 (13.4–41.3)	29.3 (9.1–49.4)	25.7 (5.0–46.4)
Hypnotherapy	28.0 (13.3–42.8)	22.6 (2.7–42.5)	33.0 (10.6–55.4)
Bupropion	27.0 (7.9–46.2)	30.1 (2.5–57.8)	24.4 (0–53.3)
None of these (unaided quitting)	21.4 (19.7–23.2)	23.2 (20.8–25.6)	18.9 (16.4–21.5)

MHC, mental health condition. NRT, nicotine replacement therapy.

Data are weighted to match the adult population in England.

^1^ Sorted by prevalence of use among all participants in the sample (highest-lowest). Note that response options were not mutually exclusive and prevalence estimates across the different aids therefore do not sum to 100%.

Corresponding data with history of MHCs coded as 0, 1, or ≥2 MHCs are provided in S2 **Table**. Data on use of cessation aids and overall quit success rates by individual MHC are provided in **S3 Table**.

**Table 2** also shows unadjusted quit success rates for those using each cessation aid. Although absolute differences appeared large for some aids (e.g., written self-help materials and nicotine pouches), wide confidence intervals meant there was no statistically significant difference in quit rates between users of aids with and without a history of mental health conditions, before adjustment for potential confounding variables and use of other aids.

**Table 3** shows the results of the logistic regression analyses. The baseline model, which included use (vs. non-use) of each cessation aid, history of mental health conditions, and covariates, indicated participants who used vaping products (OR = 1.92, 95%CI 1.61–2.30), varenicline (OR = 1.88, 95%CI 1.19–2.98), or heated tobacco products (OR = 2.33, 95%CI 1.01–5.35) in their quit attempt had significantly higher odds of quitting successfully than those who did not. Those who used Allen Carr’s Easyway method (either via face-to-face session [19.8%], book [66.9%], or both [13.3%]) had significantly lower odds of quitting successfully than those who did not (OR = 0.41, 95%CI 0.18–0.94). Use of other aids was not significantly associated with odds of quit success, after adjustment.

**Table 3:** Real-world effectiveness of cessation aids for successful smoking cessation and interactions with the user’s history of mental health conditions.

	Main effect of aid use^1^		Interaction between aid use and history of MHCs (≥1 vs. 0 MHCs)^2^			
Use in the most recent quit attempt of…	OR (95% CI)	*p*	OR (95% CI)	*p*	BF, less effective (OR = 0.67)^3^	BF, more effective (OR = 1.5)^3^
Vaping products	1.92 (1.61–2.30)	<0.001	1.25 (0.88–1.78)	0.213	0.20	1.34
NRT available over-the-counter	1.24 (0.97–1.58)	0.088	0.92 (0.57–1.48)	0.726	0.67	0.41
Prescription NRT	1.22 (0.79–1.90)	0.372	0.50 (0.20–1.21)	0.124	2.13	0.38
Varenicline	1.88 (1.19–2.98)	0.007	1.14 (0.47–2.77)	0.772	0.65	0.87
Websites	1.23 (0.74–2.05)	0.428	1.46 (0.50–4.24)	0.487	0.60	1.16
Face-to-face behavioural support	1.08 (0.59–2.00)	0.805	0.81 (0.24–2.77)	0.740	0.98	0.73
Allen Carr’s Easyway	0.41 (0.18–0.94)	0.034	0.69 (0.13–3.66)	0.665	1.06	0.78
Written self-help materials	0.50 (0.19–1.34)	0.166	0.54 (0.09–3.34)	0.510	1.06	0.75
Nicotine pouches	1.08 (0.52–2.24)	0.838	2.36 (0.53–10.5)	0.261	0.61	1.43
Telephone support	1.51 (0.64–3.57)	0.353	0.70 (0.14–3.51)	0.659	1.05	0.77
Heated tobacco products	2.33 (1.01–5.35)	0.047	0.46 (0.09–2.50)	0.371	1.26	0.68
Hypnotherapy	0.82 (0.37–1.81)	0.623	1.89 (0.37–9.71)	0.448	0.71	1.20
Bupropion	1.59 (0.45–5.68)	0.474	0.76 (0.07–8.79)	0.827	1.01	0.90

BF, Bayes factor. MHC, mental health condition. NRT, nicotine replacement therapy.

Data are weighted to match the adult population in England.

^1^ Baseline model, adjusted for use of other cessation aids, history of MHCs (0 vs. ≥1), age, gender, occupational social grade, strength of urges to smoke, time since the most recent quit attempt started, number of past-year quit attempts, whether the quit attempt was planned, whether the quit attempt was abrupt or gradual, and survey month and year.

^2^ Baseline model with the addition of the two-way interaction between use of the aid of interest and history of MHCs (0 vs. ≥1). An OR <1 indicates the aid is less effective for people with a history of MHCs than those without, and an OR >1 indicates the aid is more effective.

^3^ Bayes factor for the two-way interaction between use of the aid of interest and history of MHCs (0 vs. ≥1), based on expected effect sizes of OR = 0.67 (aid is less effective for users with a history of MHCs) and OR = 1.5 (aid is more effective for users with a history of MHCs). BFs ≥3 can be interpreted as evidence for the alternative hypothesis (i.e., effectiveness of the aid differs according to the user’s history of MHCs), BFs ≤1/3 can be interpreted as evidence for the null hypothesis (i.e., effectiveness of the aid does not differ by the user’s history of MHCs s), and BFs between 1/3 and 3 suggest that the data are insensitive to distinguish the alternative hypothesis from the null.

Corresponding data with history of MHCs coded as 0, 1, or ≥2 MHCs are provided in **S4 Table**.

Tests of interactions showed no statistically significant moderating effect of history of mental health conditions on the effectiveness of any cessation aid (**Table 3**). Bayes factors indicated the data were largely insensitive to distinguish between evidence of moderation and no evidence of moderation, meaning we were unable to rule out potential differences in effectiveness by history of mental health conditions. The only exception was for vaping products, where the data favoured the null (Bayes factor = 0.20), indicating that the effectiveness of vaping products for smoking cessation did not differ significantly between those with and without a history of mental health conditions.

There was no notable difference in the pattern of results when history of mental health conditions was analysed as a 3-level variable (distinguishing between those with none, a single mental health condition, and multiple conditions; **S4 Table**).

## Discussion

Nine in every 20 people who attempted to quit smoking had a history of one or more mental health conditions. Those with a history of mental health conditions were more likely to support their quit attempt with the use of cessation aids. Specifically, they were more likely than those without a history of mental health conditions to report using vaping products, prescription NRT, or websites, with no significant differences between groups in the use of other aids. After we adjusted for covariates and participants’ use of other cessation aids, those who used vaping products, varenicline, or heated tobacco products had significantly higher odds of quitting successfully than those who did not report using these aids. There was little evidence of benefits of using other cessation aids, or that the user’s history of mental health conditions moderated the effectiveness of any aid.

Our results echo the findings of the EAGLES trial [38], which found varenicline, bupropion, and nicotine patch were similarly effective for smokers with and without psychiatric disorders, under trial conditions. They also extend existing evidence by covering the whole range of cessation aids used by people who smoke in England and using observational data from people using these aids in the real world, where people do not necessarily receive continued monitoring and support from healthcare professionals.

The real-world effectiveness of varenicline and vaping products are established, having been reported in a number of previous studies [15–24]. In 2019, we used data from the Smoking Toolkit Study to examine the effectiveness of different cessation aids when used in real-world settings [23]. Our results suggested using varenicline or vaping products was associated with the highest odds of success in a quit attempt. When we controlled for use of other aids and other potential confounding variables (e.g., level of dependence), we found that people who used varenicline or vaping products in a quit attempt had 1.82- and 1.95-times higher odds, respectively, of remaining abstinent than those who did not. Our present results, which use data from the same survey but over a different time frame and with additional adjustment for history of mental health conditions, are consistent with these estimates.

However, to our knowledge, this is the first observational study to examine the effectiveness of heated tobacco products, because use of these products until recently has been rare in England [47]. We documented an association between use of heated tobacco products and increased odds of quit success [30]. Our data indicate use of these products in quit attempts remains relatively rare (consistent with low overall prevalence of use among adults in England [47]). As a result, the 95% CI around the estimate was wide and included the possibility of no meaningful difference (lower CI = 1.01), so conclusions may change with more data. It will be important to continue to monitor the effectiveness of heated tobacco products as the number of people using them to quit smoking grows.

The lack of evidence for differential effectiveness of any cessation aid by the user’s history of mental health conditions should provide reassurance to people with mental health conditions who want to stop smoking that their condition need not affect their choice of cessation aid. Of note, vaping products were both the most popular aid used by people with and without a history of mental health conditions and one of the most effective. Vaping products were also the only aid for which the data provided clear evidence that effectiveness was not lower for users with a history of mental health conditions (data for the other aids were insensitive).

Our results also suggest that healthcare professionals can base their recommendations for, and prescription of, smoking cessation treatments to people with mental health conditions on evidence of their effectiveness in the general population. Previous research has shown that people with mental health conditions have lower odds of being prescribed varenicline than NRT, despite having greater odds of quitting successfully with varenicline than NRT [54]. Consistent with this, our data show higher prevalence of use of prescription NRT among people with a history of mental health conditions than those without, and significantly higher odds of quit success among users of varenicline, but not NRT. Healthcare professionals may opt to prescribe NRT over varenicline for patients with mental health conditions due to concerns that varenicline may increase the risk of neuropsychiatric adverse events [55]. However, much evidence of this risk is based on information contained in case reports [55], and a large RCT of the relative neuropsychiatry safety of varenicline compared with nicotine patch and placebo among people with and without psychiatric disorders observed no significant increase in adverse events among those randomised to use varenicline [38]. If the risk of adverse events are similar, offering the more effective treatment (varenicline, once available again) is likely to be the better option.

This study had several limitations. First, questions on mental health conditions relied on self-reports, which may be less accurate than if linked health record data were used. Second, the items assessed ever (as opposed to current) diagnoses. As such, the results cannot tell us whether treatment effectiveness differs according to the user’s current mental health status. In addition, those with a history of multiple mental health conditions may also have been diagnosed with these conditions at different points in time, so a history of multiple mental health conditions may not reflect current comorbidity. Third, to boost statistical power for analyses, we grouped together participants reporting any of the mental health conditions we assessed. As the list of conditions was heterogeneous, covering a broad range of conditions from common mental health disorders (e.g., anxiety and depression) to severe mental illness (psychosis), we cannot rule out the possibility that certain conditions may moderate the effectiveness of cessation aids while others do not. Given the low prevalence of most of these conditions within the general population, much larger samples would be required to explore this. Fourth, despite combining all mental health conditions, Bayes factors indicated our data were insensitive to distinguish between evidence of absence of an interaction between aid use and mental health conditions (i.e., mental health conditions moderate treatment effectiveness) and absence of evidence for the majority of aids (all except vaping products). This means we are unable to conclusively rule out there being small to moderate differences in effectiveness by people’s history of mental health conditions. Fifth, although we adjusted for a range of potential confounders, there may be residual confounding by other variables not included in our models. Finally, it is possible that smoking cessation treatments offered to people with mental health conditions may differ from the general population, introducing potential selection bias from the treatment provider in addition to the user. Although clinical trials suggest varenicline and bupropion are effective for people with severe mental illness [38, 56], in the real world, people with these conditions are rarely offered these medications by clinicians [54]. Interactions with other medications is an important consideration in deciding on a treatment approach: bupropion is known to interact with other medications (including those indicated for treatment of mental health conditions), which may reduce the effectiveness of one or both treatments [57]. Nonetheless, our data provide useful insights into potential differences in the effectiveness of popular smoking cessation treatments in a real-world setting.

In conclusion, use of vaping products, varenicline, or heated tobacco products in a quit attempt was associated with significantly greater odds of successful cessation, after adjustment for use of other cessation aids and potential confounders. There was no evidence to suggest the effectiveness of any popular cessation aid differed according to the user’s history of mental health conditions.

## Supporting information

S1 Table Weighted sample characteristics– 3-level mental health variable. (PDF)

S2 Table Use of cessation aids in the most recent quit attempt by history of mental health conditions– 3-level mental health variable. (PDF)

S3 Table Use of cessation aids in the most recent quit attempt by history of mental health conditions–by individual mental health condition. (PDF)

S4 Table Real-world effectiveness of cessation aids for success in stopping smoking and interactions with the user’s history of mental health conditions– 3-level mental health variable. (PDF)

S1 Questionnaire Smoking Toolkit Study questionnaire. (DOCX)

## References

[1] NHS Digital. Statistics on Smoking, England 2020. In: NHS Digital [Internet]. 2020 [cited 23 Jun 2022]. Available: https://digital.nhs.uk/data-and-information/publications/statistical/statistics-on-smoking/statistics-on-smoking-england-2020

[2] Taylor E, Brose LS, McNeill A, Brown J, Kock L, Robson D. Associations between smoking and vaping prevalence, product use characteristics, and mental health diagnoses in Great Britain: a population survey. BMC Med. 2023;21: 211. doi: 10.1186/s12916-023-02890-y 37316913 PMC10268384

[3] Brose LS, Brown J, Robson D, McNeill A. Mental health, smoking, harm reduction and quit attempts–a population survey in England. BMC Public Health. 2020;20: 1237. doi: 10.1186/s12889-020-09308-x 32795286 PMC7427923

[4] Richardson S, McNeill A, Brose L. Smoking and quitting behaviours by mental health conditions in Great Britain (1993–2014). Addict Behav. 2019;90: 14–19. doi: 10.1016/j.addbeh.2018.10.011 30352340 PMC6334164

[5] Royal College of Physicians, Royal College of Psychiatrists. Smoking and mental health. London: Royal College of Physicians; 2013. Available: https://www.rcplondon.ac.uk/projects/outputs/smoking-and-mental-health

[6] De Hert M, Correll CU, Bobes J, Cetkovich-Bakmas M, Cohen D, Asai I, et al. Physical illness in patients with severe mental disorders. I. Prevalence, impact of medications and disparities in health care. World Psychiatry. 2011;10: 52–77. doi: 10.1002/j.2051-5545.2011.tb00014.x 21379357 PMC3048500

[7] Nielsen RE, Banner J, Jensen SE. Cardiovascular disease in patients with severe mental illness. Nat Rev Cardiol. 2021;18: 136–145. doi: 10.1038/s41569-020-00463-7 33128044

[8] Chang C-K, Hayes RD, Perera G, Broadbent MTM, Fernandes AC, Lee WE, et al. Life expectancy at birth for people with serious mental illness and other major disorders from a secondary mental health care case register in London. PloS One. 2011;6: e19590. doi: 10.1371/journal.pone.0019590 21611123 PMC3097201

[9] Chesney E, Robson D, Patel R, Shetty H, Richardson S, Chang C-K, et al. The impact of cigarette smoking on life expectancy in schizophrenia, schizoaffective disorder and bipolar affective disorder: An electronic case register cohort study. Schizophr Res. 2021;238: 29–35. doi: 10.1016/j.schres.2021.09.006 34563995 PMC8653908

[10] Pirie K, Peto R, Reeves GK, Green J, Beral V. The 21st century hazards of smoking and benefits of stopping: a prospective study of one million women in the UK. The Lancet. 2013;381: 133–141. doi: 10.1016/S0140-6736(12)61720-6 23107252 PMC3547248

[11] Hartmann‐Boyce J, Chepkin SC, Ye W, Bullen C, Lancaster T. Nicotine replacement therapy versus control for smoking cessation. Cochrane Database Syst Rev. 2018 [cited 8 Nov 2018]. doi: 10.1002/14651858.CD000146.pub5 29852054 PMC6353172

[12] Hartmann-Boyce J, Lindson N, Butler AR, McRobbie H, Bullen C, Begh R, et al. Electronic cigarettes for smoking cessation. Cochrane Database Syst Rev. 2022 [cited 4 Jan 2023]. doi: 10.1002/14651858.CD010216.pub7 33052602 PMC8094228

[13] Hartmann-Boyce J, Livingstone-Banks J, Ordóñez-Mena JM, Fanshawe TR, Lindson N, Freeman SC, et al. Behavioural interventions for smoking cessation: an overview and network meta‐analysis. Cochrane Database Syst Rev. 2021 [cited 30 Sep 2021]. doi: 10.1002/14651858.CD013229.pub2 33411338 PMC11354481

[14] Livingstone-Banks J, Fanshawe TR, Thomas KH, Theodoulou A, Hajizadeh A, Hartman L, et al. Nicotine receptor partial agonists for smoking cessation. Cochrane Database Syst Rev. 2023 [cited 28 Jul 2023]. doi: 10.1002/14651858.CD006103.pub8 37142273 PMC10169257

[15] Brown J, Beard E, Kotz D, Michie S, West R. Real-world effectiveness of e-cigarettes when used to aid smoking cessation: a cross-sectional population study. Addiction. 2014;109: 1531–1540. doi: 10.1111/add.12623 24846453 PMC4171752

[16] Kotz D, Brown J, West R. ‘Real-world’effectiveness of smoking cessation treatments: a population study. Addiction. 2014;109: 491–499. doi: 10.1111/add.12429 24372901

[17] Kotz D, Brown J, West R. Prospective cohort study of the effectiveness of smoking cessation treatments used in the “real world.” Mayo Clinic Proceedings. Elsevier; 2014. pp. 1360–1367. 10.1016/j.mayocp.2014.07.004 PMC4194355 25282429

[18] Chaiton M, Diemert LM, Bondy SJ, Cohen JE, Fung MD, Zhang BR, et al. Real-World Effectiveness of Pharmaceutical Smoking Cessation Aids: Time-Varying Effects. Nicotine Tob Res. [cited 13 Oct 2018]. doi: 10.1093/ntr/nty194 30260455

[19] Benmarhnia T, Pierce JP, Leas E, White MM, Strong DR, Noble ML, et al. Can e-Cigarettes and Pharmaceutical Aids Increase Smoking Cessation and Reduce Cigarette Consumption? Findings from a Nationally Representative Cohort of American Smokers. Am J Epidemiol. 2018. doi: 10.1093/aje/kwy129 29955810 PMC6211241

[20] Taylor GM, Taylor AE, Thomas KH, Jones T, Martin RM, Munafò MR, et al. The effectiveness of varenicline versus nicotine replacement therapy on long-term smoking cessation in primary care: a prospective cohort study of electronic medical records. Int J Epidemiol. 2017;46: 1948–1957. doi: 10.1093/ije/dyx109 29040555 PMC5837420

[21] Smith PH, Kasza KA, Hyland A, Fong GT, Borland R, Brady K, et al. Gender Differences in Medication Use and Cigarette Smoking Cessation: Results From the International Tobacco Control Four Country Survey. Nicotine Tob Res. 2015;17: 463–472. doi: 10.1093/ntr/ntu212 25762757 PMC4402353

[22] Yong H-H, Hitchman SC, Cummings KM, Borland R, Gravely SML, McNeill A, et al. Does the Regulatory Environment for E-Cigarettes Influence the Effectiveness of E-Cigarettes for Smoking Cessation?: Longitudinal Findings From the ITC Four Country Survey. Nicotine Tob Res. 2017;19: 1268–1276. doi: 10.1093/ntr/ntx056 28340053 PMC5896424

[23] Jackson SE, Kotz D, West R, Brown J. Moderators of real-world effectiveness of smoking cessation aids: a population study. Addiction. 2019;114: 1627–1638. doi: 10.1111/add.14656 31117151 PMC6684357

[24] Jackson SE, Kock L, Kotz D, Brown J. Real-world effectiveness of smoking cessation aids: A population survey in England with 12-month follow-up, 2015–2020. Addict Behav. 2022;135: 107442. doi: 10.1016/j.addbeh.2022.107442 35908322 PMC9587352

[25] Borland R, Li L, Driezen P, Wilson N, Hammond D, Thompson ME, et al. Cessation assistance reported by smokers in 15 countries participating in the International Tobacco Control (ITC) policy evaluation surveys. Addict Abingdon Engl. 2012;107: 197–205. doi: 10.1111/j.1360-0443.2011.03636.x 21883605 PMC3237953

[26] Pfizer Ltd. UK Supply Update–Champix (Varenicline Tartrate). 2021. Available: https://www.ncsct.co.uk/usr/pub/Champix%20Supply%20Statement%2025.6.21.pdf

[27] National Centre for Smoking Cessation and Training. Bupropion (Zyban) supply disruption: update and guidance on alternatives for clients. 2022 Dec. Available: https://www.ncsct.co.uk/usr/pub/Zyban%20supply%20disruption%20-%20update%20and%20guidance%20on%20alternatives%20for%20clients.pdf

[28] Medicines and Healthcare products Regulatory Agency. MHRA Products | Product results. [cited 15 Jun 2022]. Available: https://products.mhra.gov.uk/product/?product=TACTIZEN%201.5%20MG%20TABLETS

[29] West R, Beard E, Kale D, Kock L, Brown J. Electronic cigarettes in England—latest trends. 2021. Available: http://www.smokinginengland.info/latest-statistics/

[30] Tattan-Birch H, Hartmann-Boyce J, Kock L, Simonavicius E, Brose L, Jackson S, et al. Heated tobacco products for smoking cessation and reducing smoking prevalence. Cochrane Database Syst Rev. 2022 [cited 21 Jul 2022]. doi: 10.1002/14651858.CD013790.pub2 34988969 PMC8733777

[31] Cancer Research UK and Action on Smoking and Health. A changing landscape: Stop smoking services and tobacco control in England. 2019. Available: https://ash.org.uk/wp-content/uploads/2019/03/2019-LA-Survey-Report.pdf

[32] Frings D, Albery IP, Moss AC, Brunger H, Burghelea M, White S, et al. Comparison of Allen Carr’s Easyway programme with a specialist behavioural and pharmacological smoking cessation support service: a randomized controlled trial. Addiction. 2020 [cited 3 Apr 2020]. doi: 10.1111/add.14897 31968400 PMC7186816

[33] Keogan S, Li S, Clancy L. Allen Carr’s Easyway to Stop Smoking—A randomised clinical trial. Tob Control. 2019;28: 414–419. doi: 10.1136/tobaccocontrol-2018-054243 30361322 PMC6589447

[34] Buss V, West R, Kock L, Kale D, Brown J. Monthly trends on smoking in England from the Smoking Toolkit Study. 2023 [cited 8 Feb 2023]. Available: https://smokinginengland.info/graphs/monthly-tracking-kpi

[35] Brose LS, Brown J, McNeill A. Mental health and smoking cessation—a population survey in England. BMC Med. 2020;18: 161. doi: 10.1186/s12916-020-01617-7 32580770 PMC7315517

[36] Tidey JW, Miller ME. Smoking cessation and reduction in people with chronic mental illness. BMJ. 2015;351: h4065. doi: 10.1136/bmj.h4065 26391240 PMC4707528

[37] Lehner RK, Dopke CA, Cohen K, Edstrom K, Maslar M, Slagg NB, et al. Outpatient Treatment Adherence and Serious Mental Illness: A Review of Interventions. Am J Psychiatr Rehabil. 2007;10: 245–274. doi: 10.1080/15487760601166324

[38] Anthenelli RM, Benowitz NL, West R, Aubin LS, McRae T, Lawrence D, et al. Neuropsychiatric safety and efficacy of varenicline, bupropion, and nicotine patch in smokers with and without psychiatric disorders (EAGLES): a double-blind, randomised, placebo-controlled clinical trial. The Lancet. 2016;387: 2507–2520. doi: 10.1016/S0140-6736(16)30272-0 27116918

[39] Zhang H, Gilbert E, Hussain S, Veldhuizen S, Le Foll B, Selby P, et al. Effectiveness of Bupropion and Varenicline for Smokers With Baseline Depressive Symptoms. Nicotine Tob Res. 2023;25: 937–944. doi: 10.1093/ntr/ntac288 36520964

[40] Benson K, Hartz AJ. A comparison of observational studies and randomized, controlled trials. N Engl J Med. 2000;342: 1878–1886. doi: 10.1056/NEJM200006223422506 10861324

[41] Fidler JA, Shahab L, West O, Jarvis MJ, McEwen A, Stapleton JA, et al. “The smoking toolkit study”: a national study of smoking and smoking cessation in England. BMC Public Health. 2011;11: 479. doi: 10.1186/1471-2458-11-479 21682915 PMC3145589

[42] Jackson SE, Beard E, Kujawski B, Sunyer E, Michie S, Shahab L, et al. Comparison of Trends in Self-reported Cigarette Consumption and Sales in England, 2011 to 2018. JAMA Netw Open. 2019;2: e1910161. doi: 10.1001/jamanetworkopen.2019.10161 31461148 PMC6716287

[43] Jackson SE, Beard E, Angus C, Field M, Brown J. Moderators of changes in smoking, drinking and quitting behaviour associated with the first COVID-19 lockdown in England. Addiction. 2022;117: 772–783. doi: 10.1111/add.15656 34431577 PMC8652848

[44] Jackson SE, Garnett C, Shahab L, Oldham M, Brown J. Association of the Covid-19 lockdown with smoking, drinking, and attempts to quit in England: an analysis of 2019–2020 data. Addiction. 2021;116: 1233–1244. doi: 10.1111/add.15295 33089562 PMC8436745

[45] Kock L, Tattan-Birch H, Jackson S, Shahab L, Brown J. Socio-demographic, smoking and drinking characteristics in GB: A comparison of independent telephone and face-to-face Smoking and Alcohol Toolkit surveys conducted in March 2022. Qeios. 2022 [cited 18 Aug 2022]. doi: 10.32388/CLXK4D

[46] Tattan-Birch H, Jackson SE, Dockrell M, Brown J. Tobacco-free Nicotine Pouch Use in Great Britain: A Representative Population Survey 2020–2021. Nicotine Tob Res. 2022; ntac099. doi: 10.1093/ntr/ntac099 35417551 PMC9356773

[47] Tattan-Birch H, Brown J, Shahab L, Jackson SE. Trends in use of e-cigarette device types and heated tobacco products from 2016 to 2020 in England. Sci Rep. 2021;11: 13203. doi: 10.1038/s41598-021-92617-x 34168216 PMC8225841

[48] West RJ, Hajek P, Belcher M. Severity of withdrawal symptoms as a predictor of outcome of an attempt to quit smoking. Psychol Med. 1989;19: 981–985. doi: 10.1017/s0033291700005705 2594893

[49] Fidler JA, Shahab L, West R. Strength of urges to smoke as a measure of severity of cigarette dependence: comparison with the Fagerström Test for Nicotine Dependence and its components. Addict Abingdon Engl. 2011;106: 631–638. doi: 10.1111/j.1360-0443.2010.03226.x 21134020

[50] Kuipers MAG, West R, Beard EV, Brown J. Impact of the “Stoptober” Smoking Cessation Campaign in England From 2012 to 2017: A Quasiexperimental Repeat Cross-Sectional Study. Nicotine Tob Res Off J Soc Res Nicotine Tob. 2020;22: 1453–1459. doi: 10.1093/ntr/ntz108 31246257 PMC7443602

[51] Brown J, Kotz D, Michie S, Stapleton J, Walmsley M, West R. How effective and cost-effective was the national mass media smoking cessation campaign ‘Stoptober’? Drug Alcohol Depend. 2014;135: 52–58. doi: 10.1016/j.drugalcdep.2013.11.003 24322004 PMC3929003

[52] Beard E, Dienes Z, Muirhead C, West R. Using Bayes factors for testing hypotheses about intervention effectiveness in addictions research. Addict Abingdon Engl. 2016;111: 2230–2247. doi: 10.1111/add.13501 27347846 PMC5111611

[53] Dienes Z. Using Bayes to get the most out of non-significant results. Front Psychol. 2014;5: 781. doi: 10.3389/fpsyg.2014.00781 25120503 PMC4114196

[54] Taylor GMJ, Itani T, Thomas KH, Rai D, Jones T, Windmeijer F, et al. Prescribing Prevalence, Effectiveness, and Mental Health Safety of Smoking Cessation Medicines in Patients With Mental Disorders. Nicotine Tob Res. 2020;22: 48–57. doi: 10.1093/ntr/ntz072 31289809 PMC7073926

[55] Purvis TL, Nelson LA, Mambourg SE. Varenicline use in patients with mental illness: an update of the evidence. Expert Opin Drug Saf. 2010;9: 471–482. doi: 10.1517/14740331003657133 20166836

[56] Beard E, Jackson SE, Anthenelli RM, Benowitz NL, Aubin LS, McRae T, et al. Estimation of risk of neuropsychiatric adverse events from varenicline, bupropion and nicotine patch versus placebo: secondary analysis of results from the EAGLES trial using Bayes factors. Addict Abingdon Engl. 2021;116: 2816–2824. doi: 10.1111/add.15440 33885203 PMC8612131

[57] Zyban 150 mg prolonged release tablets—Summary of Product Characteristics (SmPC). In: medicines.org.uk [Internet]. [cited 4 Sep 2023]. Available: https://www.medicines.org.uk/emc/product/3827/smpc/print

