# Supplementary material for: Moderation of the real-world effectiveness of smoking cessation aids by mental health conditions: A population study
Source: PLOS Ment Health. 2024 Jun 4;1(1):e0000007. doi: 10.1371/journal.pmen.0000007 (PMC12798440; doi:10.1371/journal.pmen.0000007)
Supplement: S1 Table — (PDF) [file pmen.0000007.s001.pdf]

S1 Table. Weighted sample characteristics – 3-level mental health variable

	No history of a MHC	Single MHC	Multiple MHCs
Unweighted N	*3069*	*950*	*1574*
**Sociodemographic characteristics**			
Age (years), %			
16-24	19.4 (17.9-21.0)	19.2 (16.7-22.0)	26.5 (24.2-28.9)
25-34	26.5 (24.7-28.3)	27.3 (24.3-30.6)	30.3 (27.9-32.9)
35-44	18.4 (16.9-20.0)	16.4 (14.0-19.1)	19.6 (17.5-21.9)
45-54	15.7 (14.4-17.1)	17.2 (14.8-19.9)	12.4 (10.8-14.1)
55-64	11.0 (10.0-12.2)	12.6 (10.6-14.9)	7.7 (6.5-9.2)
≥65	9.0 (8.1-10.0)	7.3 (5.8-9.2)	3.5 (2.7-4.5)
Sex, %			
Man	59.8 (57.9-61.7)	49.0 (45.6-52.4)	39.2 (36.6-41.9)
Woman	40.0 (38.1-41.8)	50.8 (47.4-54.2)	58.4 (55.7-61.0)
In another way[1]	0.2 (0.1-0.5)	0.2 (0-0.8)	2.4 (1.7-3.2)
Social grade, %			
ABC1 (more advantaged)	46.0 (44.1-47.8)	42.7 (39.4-46.0)	38.1 (35.7-40.6)
C2DE (less advantaged)	54.0 (52.2-55.9)	57.3 (54.0-60.6)	61.9 (59.4-64.3)
**Level of cigarette addiction**			
Strength of urges to smoke, mean (SD)	1.5 (1.2)	1.7 (1.3)	1.8 (1.3)
**Features of the most recent quit attempt**			
Time since quit attempt started, %			
< 1 month	16.0 (14.6-17.4)	13.8 (11.7-16.3)	15.8 (13.9-17.9)
Between 1 and 6 months	45.5 (43.6-47.4)	49.1 (45.6-52.5)	47.0 (44.3-49.6)
> 6 months	38.5 (36.7-40.4)	37.1 (33.8-40.5)	37.3 (34.7-39.9)
Number of past-year quit attempts, %			
1	65.0 (63.1-66.8)	64.7 (61.3-67.9)	64.2 (61.6-66.7)
2	20.3 (18.8-21.9)	20.4 (17.7-23.3)	19.9 (17.9-22.1)
3	7.2 (6.3-8.3)	7.6 (6.0-9.6)	8.6 (7.2-10.2)
≥4	7.5 (6.5-8.6)	7.4 (5.8-9.3)	7.3 (6.0-8.8)
Quit attempt was unplanned, %	56.4 (54.5-58.4)	56.3 (52.8-59.7)	57.0 (54.3-59.6)
Quit attempt was abrupt, %	53.1 (51.1-55.0)	56.5 (53.1-59.9)	52.2 (49.5-54.9)

MHC, mental health condition. Data are weighted to match the adult population in England.
Note: There were some missing data for the following variables: age *n*=3, sex *n*=9, strength of urges to smoke *n*=77, time since quit attempt started *n*=69, quit attempt was unplanned *n*=179, quit attempt was abrupt *n*=80. Valid percentages are shown.
[1] This group was excluded from the regression analyses (which adjust for sex) in **S4 Table** due to low numbers.
