# Supplementary material for: Moderation of the real-world effectiveness of smoking cessation aids by mental health conditions: A population study
Source: PLOS Ment Health. 2024 Jun 4;1(1):e0000007. doi: 10.1371/journal.pmen.0000007 (PMC12798440; doi:10.1371/journal.pmen.0000007)
Supplement: S2 Table — (PDF) [file pmen.0000007.s002.pdf]

S2 Table. Use of cessation aids in the most recent quit attempt by history of mental health conditions – 3-level mental health variable

	% (95% CI)		
	No history of a MHC	Single MHC	Multiple MHCs
**Use in the most recent quit attempt of…[1]**			
Vaping products	30.7 (28.9–32.5)	36.7 (33.4–40.0)	40.0 (37.4–42.6)
NRT available over-the-counter	17.8 (16.4–19.3)	15.7 (13.2–18.2)	17.2 (15.2–19.3)
Prescription NRT	2.7 (2.1–3.3)	5.0 (3.6–6.5)	4.7 (3.6–5.8)
Varenicline	3.5 (2.8–4.3)	4.1 (2.8–5.4)	3.0 (2.1–3.9)
Websites	2.2 (1.6–2.7)	3.0 (1.9–4.2)	4.6 (3.4–5.7)
Face-to-face behavioural support	1.8 (1.3–2.3)	2.3 (1.3–3.3)	3.0 (2.2–3.9)
Allen Carr's Easyway	1.2 (0.7–1.6)	1.2 (0.4–1.9)	1.5 (0.8–2.1)
Written self-help materials	1.1 (0.6–1.5)	0.4 (0–0.8)	0.9 (0.4–1.4)
Nicotine pouches	0.8 (0.4–1.1)	0.9 (0.3–1.5)	1.1 (0.6–1.7)
Telephone support	0.7 (0.4–1.0)	0.8 (0.2–1.3)	1.1 (0.5–1.6)
Heated tobacco products	0.7 (0.3–1.0)	0.7 (0.2–1.2)	1.0 (0.5–1.5)
Hypnotherapy	0.6 (0.3–0.9)	0.9 (0.2–1.5)	0.7 (0.3–1.1)
Bupropion	0.4 (0.2–0.6)	0.7 (0.2–1.1)	0.5 (0–1.0)
None of these (unaided quitting)	44.4 (43.0–45.8)	42.9 (39.5–46.3)	40.1 (37.5–42.7)
**Quit success among those who used…**			
Vaping products	24.8 (21.7–27.8)	28.8 (23.7–33.9)	26.6 (22.8–30.4)
NRT available over-the-counter	19.2 (15.6–22.8)	20.1 (13.2–27.1)	21.0 (15.7–26.3)
Prescription NRT	21.6 (10.5–32.8)	18.8 (7.1–30.5)	21.7 (11.5–31.9)
Varenicline	21.5 (12.0–30.9)	29.2 (13.8–44.7)	17.3 (6.1–28.5)
Websites	20.4 (9.5–31.3)	23.0 (6.2–39.9)	32.4 (19.6–45.1)
Face-to-face behavioural support	20.0 (8.0–32.0)	34.7 (11.6–57.8)	20.6 (7.4–33.7)
Allen Carr's Easyway	13.3 (1.7–25.0)	7.2 (0–22.9)	22.0 (1.3–42.7)
Written self-help materials	10.9 (0–23.5)	0 (0–0)	26.0 (1.2–50.7)
Nicotine pouches	18.2 (1.7–34.7)	26.0 (0–63.4)	32.5 (6.0–58.9)
Telephone support	36.4 (12.6–60.1)	23.5 (0–61.0)	28.9 (4.4–53.3)
Heated tobacco products	29.3 (9.1–49.4)	30.9 (0–77.6)	23.4 (0–47.6)
Hypnotherapy	22.6 (2.7–42.5)	31.2 (0–69.9)	34.3 (3.5–65.2)
Bupropion	30.1 (2.5–57.8)	27.1 (0–66.2)	22.3 (0–77.1)
None of these (unaided quitting)	21.4 (19.7–23.2)	20.2 (15.9–24.6)	18.1 (14.9–21.3)

MHC, mental health condition. NRT, nicotine replacement therapy.
Data are weighted to match the adult population in England.
[1] Sorted by prevalence of use among all participants in the sample (highest-lowest).
