# Supplementary material for: Moderation of the real-world effectiveness of smoking cessation aids by mental health conditions: A population study
Source: PLOS Ment Health. 2024 Jun 4;1(1):e0000007. doi: 10.1371/journal.pmen.0000007 (PMC12798440; doi:10.1371/journal.pmen.0000007)
Supplement: S3 Table — (PDF) [file pmen.0000007.s003.pdf]

S3 Table. Use of cessation aids in the most recent quit attempt by history of mental health conditions – by individual mental health condition

	% (95% CI)				
	Depression	Anxiety	OCD	Panic disorder or phobia	PTSD
Unweighted N	*1803*	*1588*	*219*	*405*	*412*
**Use in the most recent quit attempt of…**[1]					
Vaping products	40.0 (37.6–42.4)	39.3 (36.7–41.9)	38.2 (31.3–45.2)	40.6 (35.6–45.7)	42.8 (37.6–48.0)
NRT available over-the-counter	17.0 (15.1–18.8)	17.4 (15.4–19.4)	20.8 (14.9–26.7)	20.4 (16.2–24.5)	19.1 (14.9–23.3)
Prescription NRT	5.0 (4.0–6.1)	5.1 (3.9–6.3)	5.2 (1.7–8.6)	7.0 (4.4–9.5)	6.1 (3.6–8.6)
Varenicline	3.6 (2.7–4.5)	3.1 (2.2–4.0)	1.1 (0–2.4)	3.3 (1.6–5.0)	3.8 (1.9–5.7)
Websites	4.0 (3.1–5.0)	4.5 (3.4–5.7)	6.0 (2.4–9.7)	5.8 (3.3–8.2)	4.5 (2.3–6.6)
Face-to-face behavioural support	2.7 (1.9–3.4)	3.0 (2.1–3.8)	3.1 (0.5–5.8)	4.8 (2.7–6.9)	4.2 (2.1–6.2)
Allen Carr's Easyway	1.3 (0.8–1.9)	1.5 (0.8–2.1)	2.1 (0–4.2)	1.7 (0.3–3.1)	1.0 (0.1–2.0)
Written self-help materials	0.7 (0.3–1.2)	0.7 (0.3–1.1)	1.1 (0–2.6)	2.0 (0.5–3.6)	1.5 (0.2–2.9)
Nicotine pouches	0.8 (0.3–1.2)	1.0 (0.5–1.5)	3.6 (0.5–6.8)	1.0 (0–1.9)	0.9 (0.1–1.8)
Telephone support	1.0 (0.5–1.5)	1.0 (0.5–1.5)	0.4 (0–1.3)	1.0 (0.1–1.9)	2.1 (0.4–3.7)
Heated tobacco products	1.0 (0.5–1.5)	0.8 (0.4–1.2)	1.3 (0–3.0)	0.9 (0–1.8)	1.4 (0.2–2.5)
Hypnotherapy	0.7 (0.3–1.1)	0.8 (0.3–1.2)	0.9 (0–2.1)	0.8 (0–1.6)	1.6 (0.3–2.8)
Bupropion	0.6 (0.2–1.1)	0.6 (0.1–1.0)	0 (0–0)	0.6 (0–1.3)	1.7 (0–3.3)
None of these (unaided quitting)	39.7 (37.2–42.1)	41.0 (38.4–43.6)	40.4 (33.3–47.4)	37.4 (32.4–42.4)	39.4 (34.3–44.5)
**Overall quit success rate**	22.4 (20.3–24.5)	22.5 (20.3–24.7)	17.2 (11.6–22.8)	17.7 (13.8–21.6)	23.8 (19.3–28.2)

ADHD, attention deficit hyperactivity disorder. ASD, autism spectrum disorder. NRT, nicotine replacement therapy. OCD, obsessive compulsive disorder. PTSD, post-traumatic stress disorder.

Data are weighted to match the adult population in England.

[1] Sorted by prevalence of use among all participants in the sample (highest-lowest).

[2] Only assessed in 2020-23.

Table continues on next page.

S3 Table. *(continued)*

	% (95% CI)				
	**Psychosis**	**Personality disorder**	**ADHD**	**Eating disorder**	**Alcohol misuse or dependence**
Unweighted N	*125*	*205*	*205*	*232*	*192*
**Use in the most recent quit attempt of…[1]**					
Vaping products	42.3 (32.7–51.8)	40.1 (32.7–47.5)	40.1 (32.7–47.5)	38.6 (31.8–45.3)	36.8 (29.5–44.1)
NRT available over-the-counter	20.6 (13.1–28.1)	18.6 (12.7–24.4)	18.6 (12.7–24.4)	16.3 (11.4–21.2)	19.9 (13.9–25.9)
Prescription NRT	7.0 (1.4–12.7)	5.7 (2.1–9.3)	5.7 (2.1–9.3)	3.1 (0.9–5.4)	8.9 (4.7–13.0)
Varenicline	0.7 (0–2.0)	2.1 (0.2–4.0)	2.1 (0.2–4.0)	2.5 (0.5–4.6)	4.8 (1.6–8.0)
Websites	3.4 (0.1–6.7)	5.5 (1.9–9.1)	5.5 (1.9–9.1)	6.0 (2.4–9.7)	4.4 (1.2–7.6)
Face-to-face behavioural support	4.4 (0.7–8.0)	6.3 (2.9–9.6)	6.3 (2.9–9.6)	4.4 (1.8–7.0)	3.1 (0.6–5.6)
Allen Carr's Easyway	1.3 (0–3.0)	1.3 (0–2.9)	1.3 (0–2.9)	1.0 (0–2.3)	1.1 (0–2.7)
Written self-help materials	0 (0–0)	1.9 (0–3.8)	1.9 (0–3.8)	2.0 (0–4.1)	2.0 (0–4.0)
Nicotine pouches	1.7 (0–5.0)	2.0 (0–4.6)	2.0 (0–4.6)	1.5 (0–3.7)	0.8 (0–2.4)
Telephone support	3.5 (0–7.6)	3.3 (0.4–6.1)	3.3 (0.4–6.1)	1.3 (0–2.6)	0.6 (0–1.8)
Heated tobacco products	0.7 (0–2.1)	0.7 (0–1.6)	0.7 (0–1.6)	0.8 (0–1.7)	1.0 (0–2.3)
Hypnotherapy	1.8 (0–4.4)	0.4 (0–1.2)	0.4 (0–1.2)	0.4 (0–1.2)	0.5 (0–1.5)
Bupropion	2.0 (0–5.8)	1.2 (0–3.5)	1.2 (0–3.5)	0.5 (0–1.5)	0 (0–0)
None of these (unaided quitting)	40.9 (31.3–50.5)	39.8 (32.5–47.1)	39.8 (32.5–47.1)	46.0 (39.0–52.9)	39.5 (32.0–46.9)
**Overall quit success rate**	13.8 (7.9–19.7)	18.4 (12.5–24.3)	18.4 (12.5–24.3)	17.8 (12.5–23.2)	22.6 (16.2–29.0)

ADHD, attention deficit hyperactivity disorder. ASD, autism spectrum disorder. NRT, nicotine replacement therapy. OCD, obsessive compulsive disorder. PTSD, post-traumatic stress disorder.
Data are weighted to match the adult population in England.
[1] Sorted by prevalence of use among all participants in the sample (highest-lowest).
[2] Only assessed in 2020-23.
Table continues on next page.

S3 Table. *(continued)*

	% (95% CI)			
	**Drug use or dependence**	**Problem gambling**	**Autism or ASD[2]**	**Bipolar disorder[2]**
Unweighted N	*219*	*33*	*113*	*91*
**Use in the most recent quit attempt of…[1]**				
Vaping products	37.4 (30.6–44.2)	38.4 (19.8–57.1)	43.1 (33.0–53.2)	46.0 (34.7–57.3)
NRT available over-the-counter	13.7 (8.7–18.6)	7.7 (0–16.9)	20.0 (11.7–28.3)	17.2 (8.4–26.1)
Prescription NRT	3.9 (1.4–6.4)	0 (0–0)	3.5 (0.4–6.6)	7.6 (0.6–14.6)
Varenicline	1.7 (0.2–3.3)	0 (0–0)	0 (0–0)	0.7 (0–2.2)
Websites	3.0 (0.9–5.0)	0 (0–0)	6.4 (1.5–11.4)	2.4 (0–5.3)
Face-to-face behavioural support	2.4 (0.5–4.3)	4.8 (0–11.8)	2.1 (0–4.6)	2.1 (0–5.2)
Allen Carr's Easyway	0.8 (0–2.0)	0 (0–0)	1.7 (0–4.1)	0 (0–0)
Written self-help materials	0 (0–0)	0 (0–0)	0 (0–0)	0.7 (0–2.2)
Nicotine pouches	1.5 (0–3.6)	0 (0–0)	0.6 (0–1.9)	2.4 (0–5.1)
Telephone support	0.9 (0–2.2)	2.4 (0–7.4)	3.7 (0.3–7.0)	3.3 (0–8.7)
Heated tobacco products	0.2 (0–0.7)	0 (0–0)	3.4 (0–7.0)	0.6 (0–1.9)
Hypnotherapy	0.7 (0–1.6)	4.9 (0–11.8)	1.2 (0–2.9)	1.7 (0–4.1)
Bupropion	0.2 (0–0.6)	0 (0–0)	0 (0–0)	2.7 (0–7.9)
None of these (unaided quitting)	45.6 (38.4–52.7)	48.7 (29.4–67.9)	44.4 (34.3–54.5)	39.1 (28.4–49.9)
**Overall quit success rate**	15.6 (10.3–20.9)	15.8 (3.8–27.8)	21.6 (13.0–30.2)	16.2 (7.8–24.7)

ADHD, attention deficit hyperactivity disorder. ASD, autism spectrum disorder. NRT, nicotine replacement therapy. OCD, obsessive compulsive disorder. PTSD, post-traumatic stress disorder.

Data are weighted to match the adult population in England.

[1] Sorted by prevalence of use among all participants in the sample (highest-lowest).

[2] Only assessed in 2020-23.
