# Supplementary material for: Moderation of the real-world effectiveness of smoking cessation aids by mental health conditions: A population study
Source: PLOS Ment Health. 2024 Jun 4;1(1):e0000007. doi: 10.1371/journal.pmen.0000007 (PMC12798440; doi:10.1371/journal.pmen.0000007)
Supplement: S4 Table — (PDF) [file pmen.0000007.s004.pdf]

S4 Table. Real-world effectiveness of cessation aids for success in stopping smoking and interactions with the user's history of mental health conditions – 3-level mental health variable

	Main effect of aid use[1]		Interaction between aid use and history of MHCs (1 vs. 0 MHCs)[2]				Interaction between aid use and history of MHCs (≥2 vs. 0 MHCs)[2]			
**Use in the most recent quit attempt of…**	**OR (95% CI)**	***p***	**OR (95% CI)**	***p***	**BF, less effective (OR=0.67)[3]**	**BF, more effective (OR=1.5)[3]**	**OR (95% CI)**	***p***	**BF, less effective (OR=0.67)[3]**	**BF, more effective (OR=1.5)[3]**
Vaping products	1.92 (1.60-2.30)	<0.001	1.34 (0.84-2.15)	0.219	0.27	1.50	1.19 (0.79-1.79)	0.409	0.28	0.93
NRT available over-the-counter	1.23 (0.97-1.57)	0.089	1.00 (0.52-1.94)	0.994	0.65	0.64	0.87 (0.50-1.51)	0.617	0.82	0.42
Prescription NRT	1.22 (0.79-1.89)	0.374	0.50 (0.16-1.62)	0.250	1.51	0.53	0.49 (0.19-1.30)	0.154	1.90	0.43
Varenicline	1.89 (1.19-2.98)	0.007	1.64 (0.60-4.49)	0.334	0.52	1.36	0.70 (0.20-2.49)	0.580	1.09	0.68
Websites	1.23 (0.74-2.04)	0.431	1.04 (0.23-4.61)	0.960	0.87	0.90	1.69 (0.55-5.24)	0.363	0.57	1.31
Face-to-face behavioural support	1.08 (0.59-2.00)	0.804	1.00 (0.21-4.80)	0.998	0.89	0.89	0.71 (0.18-2.70)	0.610	1.07	0.71
Allen Carr's Easyway	0.41 (0.18-0.94)	0.034	0.73 (0.06-8.74)	0.800	1.01	0.90	0.67 (0.11-4.33)	0.678	1.06	0.81
Written self-help materials	0.50 (0.18-1.33)	0.165	0 (0-0)[4]	<0.001	-	-	0.75 (0.12-4.85)	0.760	1.01	0.84
Nicotine pouches	1.08 (0.52-2.24)	0.838	2.48 (0.33-18.3)	0.375	0.73	1.23	2.28 (0.50-10.5)	0.288	0.63	1.37
Telephone support	1.51 (0.64-3.57)	0.351	0.21 (0.04-1.05)	0.057	2.03	0.51	1.55 (0.23-10.3)	0.652	0.81	1.07
Heated tobacco products	2.33 (1.01-5.36)	0.047	0.50 (0.05-4.80)	0.545	1.11	0.81	0.44 (0.08-2.53)	0.358	1.27	0.69
Hypnotherapy	0.82 (0.37-1.81)	0.621	3.10 (0.30-32.5)	0.346	0.76	1.23	1.38 (0.21-9.20)	0.738	0.84	1.03
Bupropion	1.59 (0.45-5.70)	0.475	2.04 (0.12-34.8)	0.624	0.87	1.07	0.34 (0.02-5.43)	0.448	1.14	0.82

MHC, mental health condition. NRT, nicotine replacement therapy.

[1] Baseline model, adjusted for use of other cessation aids, history of MHCs (0, 1, or ≥2), age, sex, occupational social grade, strength of urges to smoke, time since the most recent quit attempt started, number of past-year quit attempts, whether the quit attempt was planned, whether the quit attempt was abrupt or gradual, and survey month and year.

[2] Baseline model with the addition of the two-way interaction between use of the aid of interest and history of MHCs (0, 1, or ≥2). An OR <1 indicates the aid is less effective for people with a history of MHCs than those without, and an OR >1 indicates the aid is more effective.

[3] Bayes factor for the two-way interaction between use of the aid of interest and history of MHCs (0 vs. ≥1), based on expected effect sizes of OR=0.67 (aid is less effective for users with a history of MHCs) and OR=1.5 (aid is more effective for users with a history of MHCs). BFs ≥3 can be interpreted as evidence for the alternative hypothesis (i.e., effectiveness of the aid differs according to the user's history of MHCs), BFs ≤1/3 can be interpreted as evidence for the null hypothesis (i.e., effectiveness of the aid does not differ by the user's history of MHCs), and BFs between 1/3 and 3 suggest that the data are insensitive to distinguish the alternative hypothesis from the null.

[4] Note: the quit success rate for participants who reported a single MHC and used written self-help materials was 0% (see **S2 Table**). This finding is likely driven by the very small sample size (just 4 participants with a single MHC reported using written self-help materials).
