# Supplementary material for: Moderation of the real-world effectiveness of smoking cessation aids by mental health conditions: A population study
Source: PLOS Ment Health. 2024 Jun 4;1(1):e0000007. doi: 10.1371/journal.pmen.0000007 (PMC12798440; doi:10.1371/journal.pmen.0000007)
Supplement: S1 Questionnaire — (DOCX) [file pmen.0000007.s005.docx]

Q632A1. Which of the following best applies to you?

Please note we are referring to cigarettes and other kinds of tobacco that you set light to and NOT electronic or 'heat-not-burn' cigarettes.

(SINGLE CODE)

1. I smoke cigarettes (including hand-rolled) every day
2. I smoke cigarettes (including hand-rolled), but not every day
3. I do not smoke cigarettes at all, but I do smoke tobacco of some kind (eg. Pipe, cigar or shisha)
4. I have stopped smoking completely in the last year
5. I stopped smoking completely more than a year ago
6. I have never been a smoker (i.e. smoked for a year or more)
7. Don’t know (DO NOT READ OUT)

IF Q632A1 = CODES 1-4 ASK Q632X5

Q632x5. How much of the time have you felt the urge to smoke in the past 24 hours?

(SINGLE CODE)

1. Not at all
2. A little of the time
3. Some of the time
4. A lot of the time
5. Almost all of the time
6. All the time
7. Don’t know (DO NOT READ OUT)

IF Q632X5 = CODES 2-6 ASK Q632X7

Q632x7. In general, how strong have the urges to smoke been?

(SINGLE CODE)

1. Slight
2. Moderate
3. Strong
4. Very strong
5. Extremely strong
6. Don’t know (DO NOT READ OUT)

IF Q632A1 = CODES 1-4 ASK Q632B7

Q632b7. How many serious attempts to stop smoking have you made in the last 12 months?
By serious attempt I mean you decided that you would try to make sure you
never smoked again. Please include any attempt that you are currently making
and please include any successful attempt made within the last year.

INTERVIEWER: IF RESPONDENT SAYS 'DON’T KNOW', ENCOURAGE THEM TO GIVE
THEIR BEST ESTIMATE

(ALLOW NUMERIC RANGE 0-150, DK)

IF Q632B7>=1

The next few questions relate to the most recent serious quit attempt
to stop smoking you made in the last 12 months ...

IF Q632B7 >=1 ASK Q632B8

Q632b8. How long ago did your most recent serious quit attempt start?
By most recent, we mean the last time you tried to quit.

(SINGLE CODE)

1. In the last week
2. More than a week and up to a month
3. More than 1 month and up to 2 months
4. More than 2 months and up to 3 months
5. More than 3 months and up to 6 months
6. More than 6 months and up to a year
7. Don’t know (DO NOT READ OUT)

IF Q632B7 >=1 ASK Q632B9

Q632b9. How long did your most recent serious quit attempt last before
you went back to smoking?

(SINGLE CODE)

1. Still not smoking
2. Less than a day
3. Less than a week
4. More than 1 week and up to a month
5. More than 1 month and up to 2 months
6. More than 2 months and up to 3 months
7. More than 3 months and up to 6 months
8. More than 6 months and up to a year
9. Don’t know (DO NOT READ OUT)

IF Q632B7 >=1 ASK Q632E40

Q632e40. Which, if any, of the following did you try to help you stop smoking
during the most recent serious quit attempt?

(MULTI CODE)

1. Nicotine replacement product (e.g. patches\gum\inhaler) without a prescription
2. Nicotine replacement product on prescription or given to you by a health professional
3. Zyban (bupropion)
4. Champix (varenicline)
5. 24. Tobacco-free nicotine pouch/pod or 'white pouches' that you place on your gum (e.g., Zyn, On!, Nordic Spirit, Velo, Lyft, Skruf)
6. Attended a Stop Smoking group
7. Attended one or more Stop Smoking one-to-one counselling\advice\support session\s
8. Phoned a Smoking Helpline
9. Visited www.nhs.uk\smokefree website
10. Visited a website other than Smokefree
11. Used an application ('app') on a handheld computer (smartphone, tablet, PDA)
12. Hypnotherapy
13. Acupuncture
14. Electronic cigarette or vaping device
15. Heat-not-burn cigarette (e.g. iQOS with HEETS, heatsticks)
16. Juul
17. Allen Carr Easyway session
18. Allen Carr Easyway book
19. The SmokeFree Formula book
20. Other book or booklet
21. Other (please specify)
22. None of these (DO NOT READ OUT)
23. Don’t know (DO NOT READ OUT)

IF Q632B7 >=1 ASK Q632C1

Q632c1. Did you cut down the amount you smoked before trying to stop completely
at your most recent serious quit attempt?

(SINGLE CODE)

1. Cut down first
2. Stopped without cutting down
3. Don’t know (DO NOT READ OUT)

IF Q632B7 >=1 ASK Q632C2

Q632c2. Which one of the following applies to your most recent serious quit attempt?

(SINGLE CODE)

1. I planned the quit for later the same day or for a date in the future
2. I started the quit attempt the moment I made the decision I was going to stop
3. Don’t know (DO NOT READ OUT)

AGE. Enter exact age

1.    <OPEN ENDED>

2.    Refused

IF AGE = 2

AGEG. Which age group applies to you?

1.    Under 16

2.    16-17

3.    18-24

4.    25-34

5.    45-54

6.    55-59

7.    60-64

8.    65-74

9.    75+

10.   Refused

GENDER. Which of the following best describes how you think of yourself?

1.  Male

2.  Female

3. In another way

The following questions are about your health. We understand that this is a highly sensitive topic and would therefore like to remind you that any information you give is strictly confidential and will be used for research purposes only. Some questions asked may not necessarily apply to you.

SY01. Since the age of 16, which of the following, if any, has a doctor or health professional ever told you that you had?

(MULTI CODE)

1. Depression
2. Anxiety
3. Obsessive Compulsive Disorder
4. Panic Disorder or a phobia
5. Post-traumatic Stress Disorder
6. Psychosis
7. Personality Disorder
8. Attention Deficit Hyperactivity Disorder
9. An Eating Disorder
10. Alcohol Misuse or Dependence
11. Drug Use or Dependence
12. Problem Gambling
13. Autism or Autism Spectrum Disorder
14. Bipolar Disorder
15. None of these
16. Don’t know (DO NOT READ OUT)
17. Prefer not to say (DO NOT READ OUT)
