# Supplementary material for: Moderation of the real-world effectiveness of smoking cessation aids by mental health conditions: A population study
Source: PLOS Ment Health. 2024 Jun 4;1(1):e0000007. doi: 10.1371/journal.pmen.0000007 (PMC12798440; doi:10.1371/journal.pmen.0000007)
Supplement: Attachment — Submitted filename: Response.docx [file pmen.0000007.s006.docx]

**Journal Requirements**

1. Please amend your detailed online Financial Disclosure statement. This is published with the article. It must therefore be completed in full sentences and contain the exact wording you wish to be published.

a) State the initials, alongside each funding source, of each author to receive each grant. For example: "This work was supported by the National Institutes of Health (####### to AM; ###### to CJ) and the National Science Foundation (###### to AM)."
b) State what role the funders took in the study. If the funders had no role in your study, please state: “The funders had no role in study design, data collection and analysis, decision to publish, or preparation of the manuscript.”

Response: Done. Our financial disclosure statement now reads:

‘This work was supported by CRUK (PRCRPG-Nov21\100002 to JB) and UK Prevention Research Partnership (MR/S037519/1 to LB). The funders had no role in study design, data collection and analysis, decision to publish, or preparation of the manuscript.’

2. Please declare all competing interests beginning with the statement "I have read the journal's policy and the authors of this manuscript have the following competing interests:"

Response: Done. Our competing interests statement now reads:

‘We have read the journal's policy and the authors of this manuscript have the following competing interests: JB has received unrestricted research funding from Pfizer and J&J, who manufacture smoking cessation medications. LS has received honoraria for talks, an unrestricted research grant and travel expenses to attend meetings and workshops from Pfizer, and has acted as paid reviewer for grant awarding bodies and as a paid consultant for health care companies. All authors declare no financial links with tobacco companies, e-cigarette manufacturers, or their representatives.’

3. We have noticed that you have uploaded Supporting Information files, but you have not included a list of legends. Please add a full list of legends for your Supporting Information files after the references list.

Response: Done

4. We do not publish any copyright or trademark symbols that usually accompany proprietary names, eg (R), (C), or TM  (e.g. next to drug or reagent names). Please remove all instances of trademark/copyright symbols throughout the text, including ® (Champix®) on page 20.

Response: Done

5. In the online submission form, you indicated that "Data are available from the corresponding author on reasonable request". All PLOS journals now require all data underlying the findings described in their manuscript to be freely available to other researchers, either 1. In a public repository, 2. Within the manuscript itself, or 3. Uploaded as supplementary information.
This policy applies to all data except where public deposition would breach compliance with the protocol approved by your research ethics board. If your data cannot be made publicly available for ethical or legal reasons (e.g., public availability would compromise patient privacy), please explain your reasons by return email and your exemption request will be escalated to the editor for approval. Your exemption request will be handled independently and will not hold up the peer review process, but will need to be resolved should your manuscript be accepted for publication. One of the Editorial team will then be in touch if there are any issues.

Response: We have now uploaded the data to Open Science Framework so they are freely available to other researchers (osf.io/5xubc).

6. Please review your reference list to ensure that it is complete and correct. If you have cited papers that have been retracted, please include the rationale for doing so in the manuscript text, or remove these references and replace them with relevant current references. Any changes to the reference list should be mentioned in the rebuttal letter that accompanies your revised manuscript. If you need to cite a retracted article, indicate the article’s retracted status in the References list and also include a citation and full reference for the retraction notice.

Response: We have checked our reference list. We confirm that it is complete and that we have not cited any retracted papers.

**Reviewer 1**

Dear Jackson et al,
It has been interesting to read the paper. I know this is well written and has important implication for addiction psychiatry and where patients with mental health disorders have comorbid Tobacco Use Disorder. However, I know we can some confounding factors like race, place of residence, migration status and educational level. Can you explain if you considered those potential confounding factors and how you addressed this issue.
Otherwise, I would accept this paper to be published.

Response: We now address the potential for residual confounding in the discussion:

‘Fifth, although we adjusted for a range of potential confounders, there may be residual confounding by other variables not included in our models.’

**Reviewer 2**

I think this is a great study, thank you for letting me review it. Just a few comments:

1. Consider replacing "stopping smoking" to "smoking cessation"
Response: We have edited ‘success in stopping smoking’ to ‘successful smoking cessation’ throughout the document.

2. Allen Carr's Easyway method is not familiar to me - consider giving an explanation for non-UK readers about what this is
Response: We have given a brief explanation and referenced other studies that have evaluated this method, which provide more information:

‘Other behavioural treatments, including hypnotherapy and Allen Carr’s Easyway method (a single-session pharmacotherapy-free behavioural programme),[32,33] are provided by private companies.’

3. Page 5 lines 89-90: However, a recent secondary analysis of another RCT suggested bupropion was less effective for smokers with depressive symptoms than those without." This is a little bit confusing. I think you should flesh out an explanation about it more
Response: We have elaborated on this to provide a clearer summary of the results of this study, in comparison with the other RCT mentioned in this paragraph:

‘One large RCT (‘EAGLES’) compared varenicline and bupropion with nicotine patch and placebo and showed similar efficacy of these medications for smokers with and without psychiatric disorders.[39] However, a recent secondary analysis of another RCT that compared the effectiveness of bupropion and varenicline reported a slightly different pattern of results.[40] While varenicline was associated with similar quitting outcomes for smokers with depressive symptoms than those without, bupropion appeared to be *less* effective as a smoking cessation aid for those with depressive symptoms.[40]’

4. There is some confusion about whether you are referring to sex or gender in your analysis. You say "women and non-binary" which would be gender but in the table it says "sex"
Response: We have edited sex to gender across all tables.

5. I think it would be awesome to see the p-values for table 2 as well as including the numbers for one type of therapy only compared to multiple, as it is unclear how many people had multiple versus just one
Response: We have added information on the number of aids used to Table 2. People with a history of mental health conditions were not only more likely to report using cessation aids, but also to report using multiple aids. We now explain this in the results text:

‘Participants with a history of mental health conditions were significantly more likely to report using one or more cessation aids (58.9%) than those without (52.9%) and to report using multiple aids (12.0% vs. 7.3%).’

We have not provided p values because this can lead to undue emphasis on differences that happen to cross an arbitrary threshold (p<0.05), which can lead to dichotomous thinking – including confusing non-significance for no difference (Principle 3 of the 2016 American Statistical Association report on statistical significance <https://amstat.tandfonline.com/doi/full/10.1080/00031305.2016.1154108#.Vt2XIOaE2MN>). Rather, we provide the 95% confidence intervals for each estimate, which allows the reader to compare the estimates across subgroups (where a non-overlap in confidence interval would be consistent with a statistically significant difference).

**Reviewer 3**

Summary
The authors present a population-based investigation in England that compares the real-world effectiveness of smoking cessation aids in patients with and without co-occurring mental health conditions.

Introduction
The Introduction draws no clear conclusion as to whether previous investigators have addressed the same question, namely “to examine whether real-world effectiveness of popular smoking cessation aids differ between users with and without a history of mental health conditions.”
• Lines 87-90 cite reference 37, concluding that a large RCT showed similar efficacy for smokers with and without psychiatric disorders.
• The Discussion also cites the EAGLES trial, with similar findings.
Are their other published studies supporting or refuting same? Since the association with psychiatric conditions constitutes the Objective of the investigation, further discussion would prove useful. Establishing whether or not the findings are a) unique and/or b) groundbreaking would significantly strengthen the presentation.
Response: We have edited this paragraph to make the extent of the evidence on this topic and contribution of our study clearer:

‘To our knowledge, just two large experimental studies have investigated whether the effectiveness of smoking cessation treatments is moderated by a person’s mental health status. These have focused on varenicline, bupropion, and NRT. One large RCT (‘EAGLES’) compared varenicline and bupropion with nicotine patch and placebo and showed similar efficacy of these medications for smokers with and without psychiatric disorders.[39] However, a recent secondary analysis of another RCT that compared the effectiveness of bupropion and varenicline reported a slightly different pattern of results.[40] While varenicline was associated with similar quitting outcomes for smokers with depressive symptoms than those without, bupropion appeared to be *less* effective for those with depressive symptoms.[40] Further research is required on these and other cessation aids. Observational data can shed light on any differences in treatment effectiveness in real-world settings.[41]’

Although the manuscript provides a statistical analysis of smoking cessation aids, the authors should consider also strengthening the investigation by characterizing the underlying epidemiologic data in England pertaining to smoking and mental conditions, ie, the percentage(s) of patients with underlying mental conditions who smoke vs those without underlying mental conditions who smoke.
Response: Associations between mental health conditions and smoking status in the Smoking Toolkit Study have been published recently in a separate paper (https://link.springer.com/article/10.1186/s12916-023-02890-y). We now make this clear in the final paragraph of the introduction:

‘Data on smoking status in relation to history of mental health conditions have been published elsewhere,[2] so this paper focused specifically on the use and effectiveness of different cessation aids among those attempting to quit smoking.’

Lines 70-90: These two paragraphs (excepting Lines 90-91) intermix two related issues that merit discussion separately.
• The first sentence of the first paragraph (Lines 70-71) references the authors’ prior work while the remainder of the paragraph transitions to findings—which are more related to cessation aids than to mental health conditions. Moving the cessation aid findings to the Discussion would strengthen the Introduction, which introduces the rationale for the investigation of mental health issues.
• Likewise, although the second paragraph (Lines 80-90) addresses mental health conditions, the content appears to blend discussion of the rationale for the investigation (appropriate to the Introduction) with additional material (more appropriate for the Discussion).
Response: We have moved the first paragraph (about our previous study’s findings) to the discussion, integrating it with the findings of the present analysis:

‘The real-world effectiveness of varenicline and vaping products are established, having been reported in a number of previous studies.[15–24] In 2019, we used data from the Smoking Toolkit Study to examine the effectiveness of different cessation aids when used in real-world settings.[23] Our results suggested using varenicline or vaping products was associated with the highest odds of success in a quit attempt. When we controlled for use of other aids and other potential confounding variables (e.g., level of dependence), we found that people who used varenicline or vaping products in a quit attempt had 1.82- and 1.95-times higher odds, respectively, of remaining abstinent than those who did not. Our present results, which use data from the same survey but over a different time frame and with additional adjustment for history of mental health conditions, are consistent with these estimates.

‘However, to our knowledge, this is the first observational study to examine the effectiveness of heated tobacco products, because use of these products until recently has been rare in England.[35] We documented an association between use of heated tobacco products and increased odds of quit success.[30] Our data indicate use of these products in quit attempts remains relatively rare (consistent with low overall prevalence of use among adults in England[35]).’

We have split the second paragraph (about potential differences in treatment effectiveness by mental health status) into two. The first outlines the rationale for why we might anticipate differences and the second summarises the existing literature on this topic. We considered moving the content of the second paragraph to the discussion, but we thought it provided important context and was useful to present it early in the paper. These paragraphs now read:

‘It is possible that effectiveness of these smoking cessation aids may differ between people with and without mental health conditions.[36] People with mental health conditions may experience stronger reinforcing effects of nicotine, more severe withdrawal symptoms when they try to quit, and greater cessation fatigue (being tired of trying to stop smoking).[37] As such, it is possible that they may benefit more from cessation aids that mimic the effect of nicotine (e.g., varenicline) or provide an alternative source of nicotine (e.g., NRT, vaping products, nicotine pouches) and less from other cessation aids (e.g., forms of behavioural support). On the other hand, people with mental health conditions may be less likely to adhere to treatments,[38] causing effectiveness to be lower.

‘To our knowledge, just two studies have investigated whether the effectiveness of smoking cessation treatments is moderated by a person’s mental health status. These have focused on varenicline, bupropion, and NRT. One large RCT (‘EAGLES’) compared varenicline and bupropion with nicotine patch and placebo and showed similar efficacy of these medications for smokers with and without psychiatric disorders.[39] However, a recent secondary analysis of another RCT that compared the effectiveness of bupropion and varenicline reported a slightly different pattern of results.[40] While varenicline was associated with similar quitting outcomes for smokers with depressive symptoms than those without, bupropion appeared to be *less* effective for those with depressive symptoms.[40] Further research is required on these and other cessation aids. Observational data can shed light on any differences in treatment effectiveness in real-world settings.[41]’

We return to it in the discussion:

‘Our results echo the findings of the EAGLES trial,[39] which found varenicline, bupropion, and nicotine patch were similarly effective for smokers with and without psychiatric disorders, under trial conditions. They also extend existing evidence by covering the whole range of cessation aids used by people who smoke in England and using observational data from people using these aids in the real world, where people do not necessarily receive continued monitoring and support from healthcare professionals.’

Lines 90-91 assert that “observational data are required to shed light on any differences in treatment effectiveness in real-world settings”. Although commonly articulated, such a perspective is not necessarily supported by the medical literature examining the topic in more detail:
A comparison of observational studies and randomized, controlled trials. New Engl J Med 2000;342:1878-86
Acknowledgment and consideration would serve to strengthen the Conclusions.
Response: We have amended the wording to ‘can shed light on’ to acknowledge that there are not always differences in estimates of treatment effectiveness, citing the NEJM study:

‘Observational data can shed light on any differences in treatment effectiveness in real-world settings.[41]’

Methods and Materials
Although the manuscript describes several individual aspects of the Smoking Toolkit study in multiple places, readers not familiar with the Toolkit (particularly those residing outside England) would benefit from a more detailed. Specifically:
• What is the overlap, if any, between the Toolkit and the reported trial?
• Lines 115-117 refer to face-to-face vs telephone interviews. What was the relationship between the national database and the interviews? Were they actually Toolkit interviews performed as part of the ongoing Toolkit survey: Or were they in addition to the Toolkit data acquisition (ie, layered on top).
• It appears that one or more of the authors may have relationships with the Toolkit effort. If so, could you please elaborate on the overlap?
I am not concerned about the propriety of such a connection; just suggesting that the reader needs a clearer picture of the interrelationships, if any.
Response: This was not a trial, but rather an observational study using data collected as part of the Smoking Toolkit Study (i.e., the Smoking Toolkit Study is the source of data for this analysis). We have clarified this in the design section of the methods:

‘This was an observational study using data from the Smoking Toolkit Study; a nationally-representative monthly cross-sectional survey of adults (≥16 years) in England.[42]’

The interviews were the sole method of data collection for the Smoking Toolkit Study. We have amended the wording to make this clear:

‘Data collection for the Smoking Toolkit Study began in November 2006 and the study continues to collect data from a new sample each month. Up to February 2020, the survey was conducted via face-to-face computer-assisted interviews. However, social distancing restrictions introduced during the Covid-19 pandemic meant that no data were collected in March 2020, and data from April 2020 onwards have been collected via telephone interviews.’

We have also added a sentence to clarify the nature of the authors’ relationship to the Smoking Toolkit Study:

‘The Smoking Toolkit Study is coordinated by this study’s authors at University College London (PI Jamie Brown).’

The methodology needs clarification of the non-contiguous time frames for the data collected.
• If the data were collected monthly between 2016-17 and 2020-23, can the authors please explain the reason for excluding the data for 2018 and 2019?
• Are the data collected monthly on an ongoing basis regardless of the time frames stated in the manuscript?
• Why did the authors choose non-contiguous time frames?
Some of these concerns may be answered by addressing the questions raised in the previous paragraph (ie, Toolkit).
Response: We have expanded on our explanation of the Smoking Toolkit Study in the design section of the methods to address these questions:

‘Data collection for the Smoking Toolkit Study began in November 2006 and the study continues to collect data from a new sample each month.’

And:

‘While a core set of questions is included in each monthly survey, other variables are only assessed in certain waves, depending on availability of competitive research funding. Questions on mental health have been collected in two periods: January 2016-December 2017 and October 2020-June 2023.’

Line 175: For those who may not be familiar with ‘Stoptober’; a short description and/or citation would prove helpful.
Response: We have added the following references for Stoptober:

- Kuipers MAG, West R, Beard EV, Brown J. Impact of the “Stoptober” Smoking Cessation Campaign in England From 2012 to 2017: A Quasiexperimental Repeat Cross-Sectional Study. Nicotine Tob Res Off J Soc Res Nicotine Tob. 2020;22: 1453–1459. doi:10.1093/ntr/ntz108
- Brown J, Kotz D, Michie S, Stapleton J, Walmsley M, West R. How effective and cost-effective was the national mass media smoking cessation campaign ‘Stoptober’? Drug Alcohol Depend. 2014;135: 52–58. doi:10.1016/j.drugalcdep.2013.11.003

Discussion
The latter portion of the manuscript, starting with the Discussion, lacks line numbers.
Response: We now provide continuous line numbers throughout the document.

The moderator of “diagnosed mental health conditions” contains a broad spectrum of conditions ranging from neuroses (mild) to psychoses (severe). While the authors conclude that the effectiveness of three cessation aids did not vary by presence or absence of the listed mental health conditions, they do not address whether additional findings might be unmasked by more detailed analysis of the nature (and implied severity) of individual underlying conditions, ie, anxiety vs psychosis. Such consideration would not only strengthen their “real world” conclusions—it might also lead to important new, yet otherwise unsuspected, findings that serve to further knowledge with regard to the study objective. Specifically, although “mental health conditions” as a group do not appear to affect the overall outcome, do any of the listed individual “mental health conditions” achieve statistical significance separately?

Response: We appreciate this suggestion. Unfortunately, given the relatively small sample sizes for the individual conditions, we lack sufficient statistical power to explore moderation by each condition separately. We raise this as a limitation in the discussion:

‘Third, to boost statistical power for analyses, we grouped together participants reporting any of the mental health conditions we assessed. As the list of conditions was heterogeneous, covering a broad range of conditions from common mental health disorders (e.g., anxiety and depression) to severe mental illness (psychosis), we cannot rule out the possibility that certain conditions may moderate the effectiveness of cessation aids while others do not. Given the low prevalence of most of these conditions within the general population, much larger samples would be required to explore this.’
